# Microalbuminuria in Children With Sickle Cell Disease in the Eastern Province of Saudi Arabia

**DOI:** 10.7759/cureus.73532

**Published:** 2024-11-12

**Authors:** Abdalla M Zayed, Sulaiman Almohaimeed, Amir Eltayeb, Hossam A Aldosari, Turki Alotaibi, Tahani Alotaibi, Rawand Alharbi, Yasser Awadallah, Shangrila Joy V Ancheta, Mohamed Alasmari, Abeer Algarni, Eman Al Ghamdi, Shadin Alamrah

**Affiliations:** 1 Pediatric Oncology, King Fahad Military Medical Complex, Dhahran, SAU; 2 Pediatric Nephrology, King Fahad Military Medical Complex, Dhahran, SAU; 3 Pediatrics, King Fahad Military Medical Complex, Dhahran, SAU; 4 Preventive Medicine, King Fahad Military Medical Complex, Dhahran, SAU; 5 Nursing, Prince Sultan Military College of Health Sciences, Dhahran, SAU; 6 College of Medicine, Imam Abdulrahman Bin Faisal University, Dammam, SAU

**Keywords:** children, eastern, microalbuminuria, saudi, sickle cell disease, southwestern

## Abstract

Background and objective: Sickle cell disease (SCD) complications, such as sickle cell nephropathy (SCN), may begin in childhood and progress insidiously to chronic kidney disease in adulthood. In Saudi Arabia (SA), there is a lack of studies evaluating kidney function in children with SCD. This study aims to assess microalbuminuria (MA) as an early marker of renal dysfunction in SCD children living in the Eastern region of SA, to potentially institute appropriate early treatment.

Materials and methods: A prospective cross-sectional study was conducted on 114 Saudi children with SCD under the age of 14 years who attended the pediatric hematology clinic for routine follow-up. Demographic and clinical information were collected from the patients and their parents, who provided informed consent. Morning urine samples were collected and tested for the presence of MA using the urinary albumin/creatinine ratio (ACR). Blood samples were also collected for basic laboratory investigations. The prevalence of MA and its correlation with various clinical and laboratory data were analyzed. Additionally, a comparison of clinical characteristics and MA was conducted between children originating from the Southwestern (SW) and Eastern regions of the country, all of whom lived in the Eastern Province.

Results: A total of 114 children with SCD were included in the study. The mean age was 8.8 ± 3.2 years, with a male-to-female ratio of 1.3:1. Based on their region of origin, they were divided into two groups: Eastern (n = 26/114) and SW (n = 88/114). MA was detected in 28 patients (24.6%), with no significant difference in prevalence between the two groups. There was no significant statistical difference in clinical and laboratory data between the groups, except for hemoglobin F (HBF) levels and the use of hydroxyurea (HU). HBF levels were significantly higher in children from the Eastern region, while more SW patients used HU. No correlation was found between MA and any of the studied variables.

Conclusion: MA is common in children with SCD in the Eastern region of SA, with no difference in its prevalence between children of the two different ancestries carrying the Arab-Indian (AI) and African haplotypes. It is not associated with any of the studied clinical variables in this report. Further studies are needed to confirm these findings.

## Introduction

Sickle cell disease (SCD) is relatively common in Saudi Arabia and is more prevalent in the Eastern and Southwestern (SW) provinces of the country [1], with genetic, clinical, and hematological variability. The clinical phenotype of SCD is heterogeneous, influenced by several genetic and environmental modifying factors, including hemoglobin F (HBF), coexistent α-thalassemia trait, and the β-globin gene cluster haplotype [2]. In the Eastern province, the sickle hemoglobin gene is typically associated with the Arab-Indian (AI) haplotype, which is linked to a milder clinical phenotype, whereas the African (Benin) haplotype predominates in the SW region and is associated with a more severe form of the disease [3].

SCD affects nearly every organ in the body [2], with kidney abnormalities, known as sickle cell nephropathy (SCN), frequently observed in children. Renal dysfunction develops slowly from childhood, with glomerular hyperfiltration (GHF) and defects in urinary concentration being the earliest manifestations. Proteinuria, impaired urinary acidification, and cortical scarring often follow, eventually leading to chronic renal failure in early adulthood [4, 5]. Microalbuminuria (MA), which occurs during the subclinical phase of SCN, appears early in life and is often followed by persistent proteinuria. MA is regarded as an early marker for glomerular dysfunction [6].

Studies on MA in patients with SCD in Saudi Arabia are limited [7-12], and its prevalence among children in the Eastern province has not been previously examined. At our hospital, patients from both the Eastern and SW regions receive medical care. Given the phenotypic variation between patients with the AI and African SCD haplotypes [3], we hypothesize that children from the SW region are more predisposed to renal damage than their Eastern counterparts. Therefore, our objective was to study the prevalence of MA and its clinical and laboratory correlates among children with SCD in the Eastern region of Saudi Arabia. We also aimed to compare the demographic, clinical, and laboratory characteristics of both groups of children with SCD living in the Eastern region of Saudi Arabia. Studying early clinical signs of renal dysfunction in children with varying SCD severity may assist in tailoring plans and allocating resources for early detection and potential preventive treatment.

## Materials and methods

A prospective cross-sectional observational study was conducted at King Fahad Military Medical Complex (KFMMC) in Dhahran, Eastern Region, Saudi Arabia, from July 2021 to August 2022.

Inclusion criteria included children younger than 14 years with homozygous SCD who attended the pediatric hematology clinic in a steady state for routine follow-up. Enrollment was completed after informed consent was obtained from their parents.

Exclusion criteria included SCD children with pre-existing renal disease and those who experienced SCD crises, acute illness, or fever within the preceding two weeks.

The following information was collected: demographic data (patients' age, place of origin, gender, age, and consanguinity) as well as clinical and laboratory findings. Place of origin (provenance) was noted to determine the patient’s ancestry and SCD haplotype, confirmed through the patient’s ID attached to their medical file. Clinical data included history of dactylitis, history of SCD complications (vaso-occlusive crisis (VOC), acute chest syndrome (ACS), splenic sequestration crisis (SSC), hemolytic crisis, stroke, cholelithiasis, and avascular necrosis of the femoral head (AVN)), family history of SCD, and use of hydroxyurea (HU). Systemic examination included measurements of weight and height with calculated body mass index (BMI), as well as systolic and diastolic blood pressure (BP). Blood pressure readings were compared with reference ranges for a pediatric cohort with SCA [13].

For the measurement of MA, early morning urine samples were collected. MA was determined by immunoturbidimetric assay, measuring the albumin/creatinine ratio (ACR) using rate nephelometry. MA was defined as an ACR of 30-300 mg albumin/g creatinine [12].

Blood samples were collected for standard laboratory parameters, including steady-state hemoglobin, hematocrit, white blood cells (WBCs), reticulocytes, platelets, hemoglobin S (HBS), HBF, serum creatinine, and lactate dehydrogenase (LDH). Hemoglobin electrophoresis was performed to confirm the SCD genotype using high-performance liquid chromatography (HPLC). The estimated glomerular filtration rate (eGFR) was calculated using the updated Schwartz equation as follows: eGFR (mL/min/1.73 m²) = 0.413 × height (cm)/serum creatinine (mg/dL) [14].

Correlation was analyzed between MA and various variables, including age, sex, BP, BMI, steady-state hemoglobin, hematocrit, WBC count, reticulocyte percentage, platelet count, HBF, serum creatinine, LDH, and hydroxyurea use.

The study was approved by the Armed Forces Hospitals Eastern Province Institutional Review Board (IRB no: AFHER-IRB-2021-018).

Statistical analysis

According to their province of origin, children were categorized into two groups: Eastern and SW. Comparisons between these groups included demographic, clinical, and laboratory data. Data were entered into Microsoft Excel 2013 (Microsoft Corporation, Redmond, Washington) and then uploaded into IBM SPSS Statistics for Windows, Version 21 (Released 2012; IBM Corp., Armonk, New York). Categorical variables were expressed as percentages, while continuous variables were expressed as means ± standard deviations. Student’s t-test was used to compare means of continuous data, and the chi-square test (χ²) was used for categorical variables. Logistic regression analysis was performed to assess the association between MA and the independent variables. A P-value of <0.05 was considered statistically significant.

## Results

The current study included 114 children with SCD (66 boys and 48 girls, male-to-female ratio of 1.3:1), with a mean age of 8.8 ± 3.2 years, ranging from 1.5 to 14 years. Among the participants, 88 patients (77.2%) were from the SW areas, and 26 patients (22.8%) were from the Eastern area. SW children are expected to carry the African beta-globin gene (HBB) cluster haplotypes, while Eastern patients are expected to have the AI haplotype, as previously described [15].

Characteristics of the patients

The demographic and clinical characteristics of the studied patients are compared and summarized in Table 1. The analysis shows no significant difference (p > 0.05) between the two groups, except that the use of hydroxyurea (HU) is significantly higher in the SW children compared with their Eastern peers. Additionally, HBF levels are significantly higher in the Eastern children compared to their SW counterparts.

**Table 1:** Comparison of the clinical characteristics of the patients according their province of origin *Significant. ^+^Patients with a complicated course include those who experienced at least one episode of SCD crises necessitating hospitalization (VOC, SSC, aplastic and hemolytic crises), stroke, ACS, GS, and AVN. M, male; F, female; FH, family history; H/O, history of; HU, hydroxyurea; BMI, body mass index; BP, blood pressure; HB, hemoglobin (steady state); Hct, hematocrit; WBCs, white blood cells; Retics, reticulocytes; PLT, platelets; HBF, hemoglobin F; LDH, lactate dehydrogenase; eGFR, estimated glomerular filtration rate; MA, microalbuminuria (ACR of 30-300 mg albumin/g creatinine); SCD: sickle cell disease, VOC: vaso-occlusive crisis, SSC: splenic sequestration crisis, ACS: acute chest syndrome, GS: gallstones, AVN: avascular necrosis.

Parameter	All patients (n = 114)	Southwest (n = 88)	East (n = 26)	P-value
Age, years	8.8±3.2	8.9±3.2	8.6±3.3	0.64
Sex				0.12
Male (n = 66)	57.9%	61.4%	46.2%	
Female (n = 48)	42.1%	38.6%	53.8%	
FH of SCD	45.6%	40.9%	61.5%	0.05
H/O dactylitis	13.2%	12.5%	15.4%	0.46
Complicated course^+^	86.8%	87.5%	84.6	0.46
HU use	61.4%	67%	42.3%	0.02*
Weight, kg	25.5±11	25.2±10.7	26.7±12	0.31
Height, cm	124.1±18.2	123.8±18.5	125.2±17.5	0.71
BMI, kg/m^2^	16±3.7	15.9±3.9	16.1±2.7	0.76
Systolic BP, mm/Hg	107±10	107±10	105±10	0.54
Diastolic BP, mm/Hg	60±8	60±8	62±9	0.31
HB, g/dL	9±1.2	8.9±1.1	9.2±1.3	0.24
Hct %	27±4	27±4	28±4	0.12
WBC × 10^9^/L	10.1±4.3	10±4.4	10.6±4.2	0.55
Retics %	7.4±4.5	7.3±4.3	7.7±5.3	0.75
PLT × 10^9^/L	390±18	399±18	359±179	0.32
HBF%	14.3±7.7	13.3±7.3	17.4±8.2	0.03*
LDH, IU/L	494±20	497±21	486±150	0.76
eGFR mL/min/1.73 m^2^	179.4±52.7	183.2±54.2	166±45	0.13
MA	24.6%	23.9%	26.9%	0.75

Prevalence of MA

**Figure 1:**
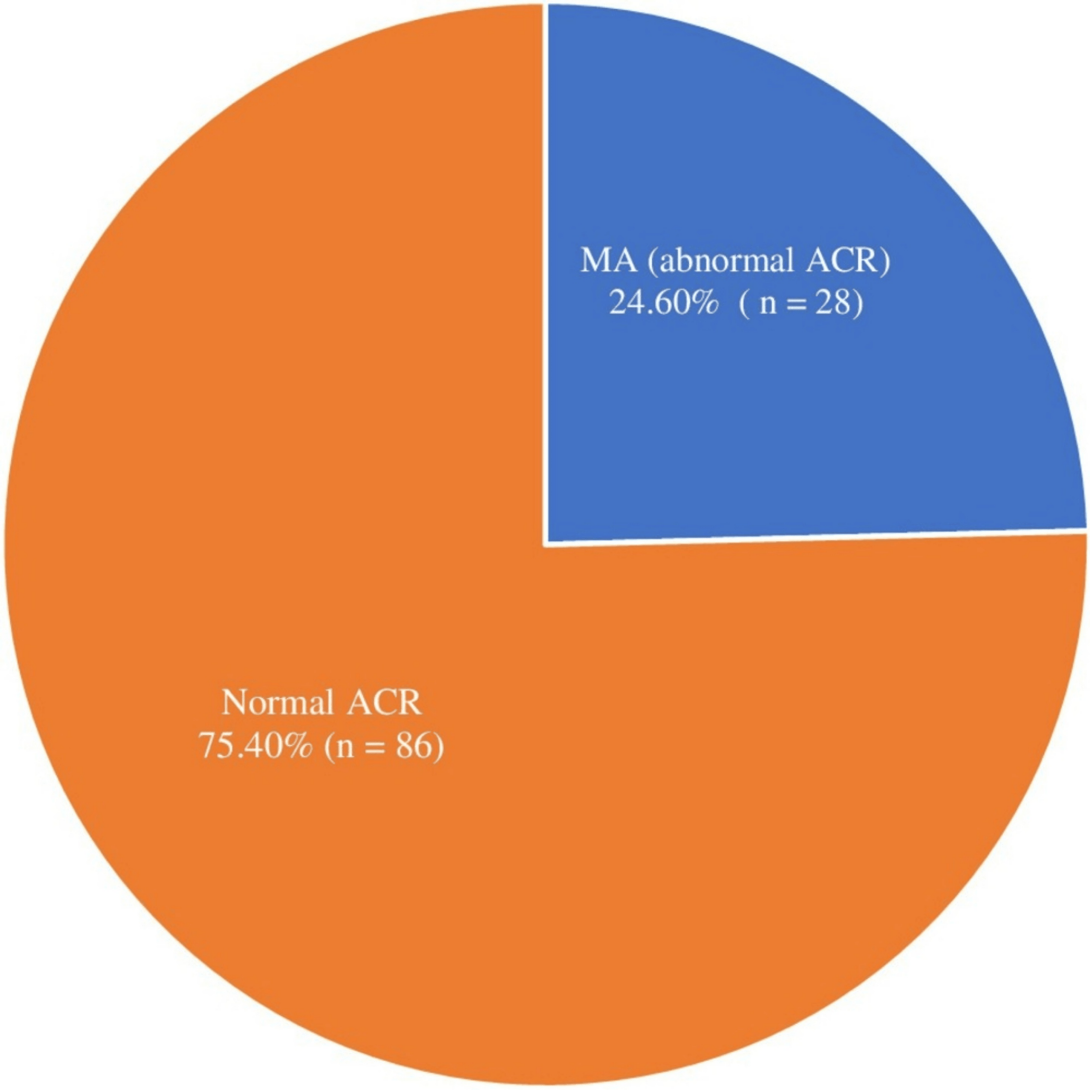
Prevalence of microalbuminuria among Saudi children with SCD MA: microalbuminuria, ACR: albumin-to-creatinine ratio, SCD: sickle cell disease.

**Figure 2:**
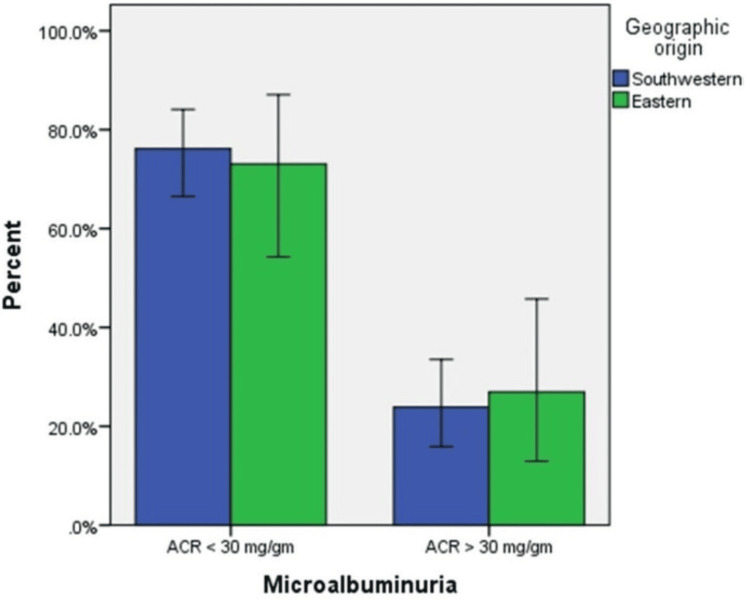
Prevalence of microalbuminuria among Eastern and Southwestern Saudi children with SCD Microalbuminuria: ACR > 30 mg/g creatinine. ACR: albumin-to-creatinine ratio, SCD: sickle cell disease.

MA was diagnosed in 28 patients, with a prevalence rate of 24.6%, as shown in Figure 1. The prevalence was 23.9% among the SW children and 26.9% among their Eastern counterparts, with no significant difference, as illustrated in Figure 2.

Comparison of the categorical variables between MA and non-MA children

Comparison of the categorical variables, including demographic and clinical data, using the chi-squared test (χ²) revealed no statistical difference between children with and without MA (Table 2). These variables included place of origin, gender, age group (less than 10 years and more than 10 years), parental consanguinity, positive family history of SCD, past history of dactylitis, use of HU, and a complicated SCD course characterized by one or more hospitalizations for VOC, ACS, SSC, stroke, aplastic or hemolytic crisis, as well as cholelithiasis and osteonecrosis. The comparison also included the prevalence of GHF between children with and without MA.

**Table 2:** Relationship between MA and different categorical and clinical parameters Complicated SCD: children who had one or more hospital admission for VOC, ACS, SSC, stroke, aplastic crisis, hemolytic crisis, gall stones, or avascular necrosis. F/H SCD: family history of SCD, H/O: history of, GHF: glomerular hyperfiltration, SCD: sickle cell disease, VOC: vaso-occlusive crisis, ACS: acute chest syndrome, SSC: splenic sequestration crisis.

Variable		Studied sample (n = 114)				Total	χ^2^ value	P-value
		MA (n = 28)		No MA (n = 86)				
Place of origin	Southwestern	21	23.9	67	76.1	88	0.101	0.750
	Eastern	7	26.9	19	73.1	26		
Gender	Male	19	28.8	47	71.2	66	1.511	0.219
	Female	9	18.8	39	81.3	48		
Age in years	Less than 10 years	15	23.4	49	76.6	64	0.099	0.752
	10 years and more	13	26.0	37	74.0	50		
Consanguinity	Present	18	23.4	59	76.6	77	0.180	0.672
	Absent	10	27.0	27	73.0	37		
F/H SCD	Positive	11	21.2	41	78.8	52	0.599	0.439
	Negative	17	27.4	45	72.6	62		
H/O dactylitis	Present	6	31.6	13	68.4	19	0.606	0.436
	Absent	22	23.2	73	76.8	95		
Hydroxyurea use	Using	16	22.9	54	77.1	70	0.284	0.594
	Not using	12	27.3	32	72.7	44		
Complicated SCD	Complicated	23	23.7	74	76.3	97	0.253	0.614
	Benign course	5	29.4	12	70.6	17		
GHF	GFR > 180 mL/min/1.73 m^2^	11	21.6	40	78.4	51	0.446	0.504
	GFR <180 mL/min/1.73 m^2^	17	27.0	46	73.0	63		

Comparison of the continuous variables between MA and non-MA children

Statistical analysis using Student's t-test for continuous variables (Table 3) did not show a significant difference between children with MA and those without in any of the studied parameters. These parameters included total hemoglobin (HB), hematocrit, WBC count, platelet count, reticulocyte percentage, HBF, urine and serum creatinine, eGFR, and LDH.

**Table 3:** Relationship between MA and different laboratory parameters HB: hemoglobin, WBC: white blood cells, LDH: lactate dehydrogenase, HBF: hemoglobin F, eGFR: estimated glomerular filtration rate.

Variables	Studied sample (n = 114)		t-value	P-value
	MA (n = 28)	No MA (n = 86)		
Total HB (g/dL)	8.983±1.17	9.049±1.23	0.24	0.80
Hematocrit %	26.436±4.94	27.892±4.04	1.56	0.12
WBC count (x 10^9^/L)	10.46±4.90	10.07±4.42	-0.37	0.71
Reticulocyte%	8.6±4.30	7.0±4.60	-1.71	0.09
Platelet count (x 10^9^/L)	420±17	391±186	-0.75	0.45
Urine creatinine (µmol/L)	4.34±2.90	5.34±3.20	1.52	0.13
Serum creatinine (µmol/L)	28.64±7.97	26.95±8.11	0.96	0.33
LDH (U/L)	509±19	490±205	-0.45	0.65
HBF (%)	12.104±7.89	14.976±7.69	1.70	0.09
eGFR (mL/min/1.73 mL)	180.4±52.80	176.4±53.1	0.34	0.72

Regression analysis

In the logistic regression analysis of MA, no correlation was found between the presence of MA and any of the studied variables. These variables included age, gender, province of origin, BMI, steady-state HB, HBF, reticulocyte percentage, eGFR, LDH, and the use of HU, as shown in Table 4.

**Table 4:** Logistic regression analysis of demographic and clinical variables on microalbuminuria BMI: body mass index, HB: hemoglobin, HBF: hemoglobin F eGFR: estimated glomerular filtration rate, H/O: history of, LDH: lactate dehydrogenase, HU: hydroxyurea.

Parameter	Odds ratio	95% Confidence interval	P-value
Age	1.01	0.88-1.15	0.87
Gender	0.57	0.23-1.40	0.22
Provenance	1.17	0.43-3.10	0.75
BMI	0.94	0.82-1.07	0.37
H/O dactylitis	1.13	0.33-3.90	0.83
Total HB	0.96	0.67-1.37	0.84
HBF	0.95	0.89-1.00	0.09
Reticulocyte%	5.33	0.90-31.55	0.06
eGFR	0.99	0.99-1.00	0.72
LDH	2.10	0.14-31.13	0.59
HU use	0.79	0.33-1.88	0.59

## Discussion

There is a paucity of research on renal dysfunction in SCD children in Saudi Arabia (Table 4), with none conducted specifically in the Eastern region of the country. Previous studies have concluded that SCD manifestations are milder in patients with the AI haplotype compared with carriers of the African haplotype [3]. Additionally, investigators from the Eastern region of Saudi Arabia have reported milder renal complications in their adult SCD patients with the AI haplotype [9]. Therefore, we hypothesize that renal impairment is less common in Eastern children with SCD. However, the prevalence of MA in this patient population has not been previously estimated.

The aim of this study was to determine the prevalence and examine the clinical correlates of MA as an early manifestation of renal dysfunction in Saudi children living in the Eastern region of Saudi Arabia.

The results indicated an overall prevalence of 24.6% among the studied patients, with no significant difference between Eastern children (with the AI haplotype) and their SW peers (with the African haplotype). Additionally, there was no correlation between MA and any of the variables studied, including age, gender, BMI, systolic or diastolic BP, steady-state HB, hematocrit, WBCs, platelets, reticulocyte percentage, HBF, LDH, SCD crises, and eGFR.

MA, with or without hyperfiltration, has been considered the earliest renal manifestation reflecting probable glomerular injury in children and adults with SCD [16]. The prevalence of MA in patients with SCD is widely variable (9.6% - 60.9%) [8, 17, 18]. In our patients, the overall prevalence was consistent with previous studies on children. An ACR of ≥30 mg/g creatinine was observed in children as young as three years old, with prevalence rates varying between 4.5% and 27% [19-21]. The prevalence and lack of any relationship between MA and studied parameters in our study are similar to the findings of Alkhunaizi et al. [9]. Both studies were cross-sectional, using the same method to assess MA, and were conducted independently in the same environment of Dhahran, Eastern Region of Saudi Arabia. However, key differences include that our patients were children, primarily originating from the SW part of the country with a predominance of the African sickle haplotype (known to be severe), whereas in Al-Khnaizi et al.’s study, the patients were adults with predominantly the AI haplotype (known to be mild). Our findings also resemble those of a Brazilian study, which could not identify any risk factors associated with proteinuria. All subjects in that study were teenagers or adults [22]. It is possible that the kidneys in this group of patients might be affected by SCD in a similar manner to Saudi patients.

The difference in MA prevalence among patients with SCD in various studies may be attributed to racial and sickle cell beta-globin haplotype differences. Additionally, variations in sample sizes, ages of the patients involved, and differences in methods used for MA screening may explain this variability [23].

In Saudi Arabia, it is known that carriers of the AI haplotype (in the East) typically experience milder disease than those carrying the African haplotype (in the SW), likely due to their higher HBF levels [3]. The distance between the two regions is approximately 1,500 kilometers, and the environmental conditions differ [15]. We hypothesized that the prevalence of MA would be higher in the SW group. However, when comparing disease characteristics between these two groups of children, we found that despite higher HBF levels in Eastern patients, there was no significant difference in the prevalence of MA. Has the experience of patients with severe disease living in an environment associated with a milder form of the disease led to some amelioration of severity, narrowing the gap between the two forms? Further studies are necessary to explore this hypothesis.

In SCD, proteinuria is believed to be age-dependent when assessed as MA (30-300 mg/g creatinine) or macro-albuminuria (>300 mg/g creatinine). MA has been reported in young children as early as 2.5 years [24]. Researchers have noted that the occurrence of MA increases with age [17, 20, 25-28]. However, Shatat et al. observed that ACR may increase or decrease over time. In limited longitudinal studies, ACR measurements have been variable; half of the patients experienced spontaneous resolution of albuminuria by the end of a three-year study [29]. Other longitudinal studies have shown that many children initially testing negative for MA converted to positive over time [30], and persistent albuminuria has been found to correlate with age [31].

The pathophysiology of SCN remains hypothetical, with chronic hemolysis-related endothelial dysfunction and relative renal hypoxia triggered by repeated vaso-occlusive crises identified as potential key factors [32]. Studies reporting relationships between MA and low levels of total HB and HBF have yielded conflicting results. Many investigators have found an association between MA and low steady-state HB [17-19, 23, 24, 28, 30, 33, 34] and low HBF [28, 29], whereas our study and others [6] did not. In our study, the results may have been influenced by the high rate of HU use (61%), which is known to increase steady-state HB and HBF levels [35]. Nonetheless, reports on the role of HU in modifying the course of SCN are mixed. There was no relationship between MA and HU use among our patients, consistent with previous studies [9, 30, 36-38].

In SA, few studies have focused on SCD patients with MA. As shown in Table 5, research from different regions of the country, representing different sickle cell beta-globin haplotypes, used varying sample sizes. All studies were cross-sectional and employed different methodologies for estimating MA. The prevalence of MA in all of them is comparable, except for one pediatric study, where the prevalence was relatively low (9.6%) and Saudis represented 50% of the study population. In two of these studies, as well as ours, there was no correlation between MA and any of the studied clinical features. However, correlations with age and low HB were observed in two studies.

**Table 5:** Saudi studies on MA in SCD patients. MA: microalbuminuria, BMI: body mass index, HB: hemoglobin, BP: blood pressure, ACS: acute chest syndrome, LDH: lactate dehydrogenase, eGFR: estimated glomerular filtration rate, DBP: diastolic blood pressure.

Reference	Prevalence of MA	Study	Relationship with MA	No relationship with MA
Our study	24.6%; 114 Children	A prospective cross-sectional observational study; Eastern Region (African > Arab-Indian) haplotype; early morning urine sample	None	Age; gender; BMI; HB; BP; pain episodes, ACS, gall stones; LDH; eGFR; hydroxyurea use
Alkhunaizi et al. (2018) [9]	25%; 72 Adult patients	A prospective cross-sectional observational study; Eastern Region (Arab-Indian) haplotype; early morning urine sample	None	Age; gender; BMI; HB; BP; pain episodes; hydroxyurea use
Aleem et al. (2010) [11]	67 Adult patients	A prospective study; Central Region (Riyadh); from the South and South-Western province; 24-hour urine	None	Age; HB; BP; HBF; LDH; GFR; hydroxyurea use
Abo-Zenah et al. (2009) [10]	133 Adult patients	Cross-sectional study (Military Hospital, Eastern Region); African haplotype; 24-hour urine	Older; more anemic; had a higher systolic BP; a lower eGFR	BMI; DBP (mm Hg)
Alzahrani et al. (2020) [8]	9.6%; 322 Children	Cross-sectional, retrospective study; Western Region; 50% non-Saudis	Acute chest syndrome; gallbladder stones	HB; age; dactylitis; osteomyelitis; stroke; avascular necrosis; splenic sequestration
Elbalawy et al. (2019) [7]	28.9%; 69 Children	Cross-sectional prospective (Military Hospital, Northern region)	Low HB; older age; males more than females	VOC; blood transfusion

The conflicting results from different studies in Saudi Arabia may be explained by variations in patient characteristics and SCD clinical features across different parts of the country. There may be a complex interaction between genetic and environmental factors in SCD patients. Larger, multicenter prospective longitudinal studies using consistent methods of MA measurement, preferably with 24-hour urine samples, would provide a more accurate assessment of MA and its correlates in this population.

Limitations of the study and future directions

The present study has some limitations, including its hospital-based cross-sectional design, which restricts the ability to make generalized conclusions. Additionally, biological measurements were performed only once during the study period. Repeated serial analyses of urine samples positive for MA over at least three months would better ensure its persistence and validity. Long-term follow-up of these patients into adulthood would be valuable in determining the true predictive value of MA in children.

## Conclusions

The prevalence of MA among children with SCD at KFMMC is 24.6%. There was no significant difference between children from the Eastern region with the AI haplotype and their peers from the SW area carrying the African haplotypes. MA was not associated with any of the studied demographic, clinical, or laboratory data. Further prospective multicenter longitudinal studies across different regions of the kingdom are needed to confirm and generalize these results.
